# Mild Cognitive Impairment in Parkinson's Disease: A Review of Current Concepts

**DOI:** 10.1155/2013/576091

**Published:** 2013-07-01

**Authors:** Natalie C. Palavra, Sharon L. Naismith, Simon J. G. Lewis

**Affiliations:** Parkinson's Disease Research Clinic, Brain & Mind Research Institute, University of Sydney, 94 Mallett Street, Camperdown, NSW 2050, Australia

## Abstract

Mild Cognitive Impairment in Parkinson's Disease (PD-MCI) is common and may be associated with accelerated progression to dementia. Considering the importance of this emerging entity, new diagnostic criteria have recently been proposed. Early recognition and accurate classification of PD-MCI could offer opportunities for novel therapeutic interventions. This review discusses current definitions for PD-MCI, the screening tools used, the pattern of cognitive deficits observed, and the predictors of cognitive decline and transition to Parkinson's Disease Dementia. Emerging biomarkers, which may aid diagnosis, are also explored and the role of novel treatment options is considered.

## 1. Introduction

Idiopathic Parkinson's Disease (PD) is a progressive neurodegenerative disorder typically characterised by its motor features: bradykinesia, tremor, rigidity, and postural instability. However, it has become increasingly apparent that nonmotor features such as cognitive impairment, constipation, bladder dysfunction, sleep disorders, depression, anxiety, and psychosis are also significant [1]. Indeed, these symptoms, which are often poorly recognised and treated, can dominate in advanced PD accounting for significant disability, impaired quality of life, and reduced life expectancy [1, 2]. 

Cognitive impairment is particularly prevalent in PD and varies from mild deficits through to severe dementia [3]. Usually, dementia is limited to the advanced stages of disease, but it affects over 80% of those with 20 years of disease [4]. By contrast, subtle cognitive impairment is common in early disease and one study has reported that over a third of patients have deficits at the time of their diagnosis [5]. Importantly, even these subtle impairments impact on quality of life [6], exacerbate caregiver distress [7], and increase the risk of nursing home placement [8]. These impairments are likely to herald the progression to dementia [9, 10] and thus the early recognition of cognitive impairment could offer a window for novel therapeutic interventions, aiming to alter the course of this natural history [11].

## 2. Defining Mild Cognitive Impairment 

In nonPD populations, Mild Cognitive Impairment (MCI) describes an intermediate stage between normal cognitive function and dementia [11], where an individual has deficits in at least one cognitive domain [12]. Diagnostic criteria have been proposed by Petersen [13], wherein MCI is characterised by a deficit of at least 1.5 standard deviations (SD) below that expected for an individual's age and education level. Unlike the presence of dementia, MCI should not impair daily functioning [12] and therefore diagnosis requires clinical interview and standardised assessment of premorbid intellectual functioning [14]. 

Original work characterising MCI in nonPD samples focused on a single deficit in the cognitive domain of memory, amnestic-MCI. Subsequently, this definition has been expanded to include deficits in domains other than memory (nonamnestic-MCI), including frontal/executive, language, attention, and visuospatial skills, and in multiple domains (multiple-MCI) [13]. It has been suggested that the existence of subtypes of MCI may represent distinct underlying pathophysiologies such as Alzheimer's Disease, vascular or frontotemporal dementia, that may have differential illness trajectories longitudinally.

## 3. Defining Mild Cognitive Impairment in Parkinson's Disease

Adopting uniform criteria for Mild Cognitive Impairment in Parkinson's Disease (PD-MCI) is critical for the identification and management of PD patients and for future therapeutic trials [15]. A previous lack of consensus criteria for PD-MCI has led to a wide range of prevalence rates due to the differing criteria employed across studies. The recent Movement Disorders Society (MDS) Task Force review reported a mean prevalence of 27%, ranging from 19% to 38% [15]. 

These disparities have prompted an MDS Task Force to propose a two-level operational schema for the diagnosis of PD-MCI [16]. Briefly, Level I diagnostic criteria include (i) a diagnosis of PD based on the UK PD Brain Bank Criteria, (ii) gradual decline in cognitive ability reported by either patient or informant, or observed by the clinician, (iii) cognitive deficits on either formal neuropsychological testing or a scale of global cognitive abilities, and (iv) cognitive deficits are not sufficient to interfere significantly with functional independence. The Level II diagnostic criteria confer greater diagnostic certainty and involve more comprehensive assessment: (i) neuropsychological testing including two tests within each of the five cognitive domains (attention and working memory, executive, language, memory, visuospatial), (ii) impairment on at least two neuropsychological tests in one cognitive domain, or one impaired test in two different cognitive domains, and (iii) impairment below appropriate norms *or* significant decline on serial cognitive testing *or *significant decline from estimated premorbid levels. Whilst such guidelines are likely to prove helpful, research is required to validate and/or refine the proposed guidelines before they are widely applied. 

## 4. Current Screening Tools for Mild Cognitive Impairment in Parkinson's Disease

Our understanding of PD-MCI is complicated by the wide variety of assessment tools utilised across different studies. Formal neuropsychological testing represents the current gold-standard, but this is time-consuming and requires specialised equipment and personnel. To optimise the diagnosis of PD-MCI in clinical practice, valid and short screening tools are necessary, and some authors have even explored the use of self-report and caregiver-report questionnaires to identify subtle cognitive deficits [17, 18]. Widely-accepted assessment tools for PD-MCI are still lacking. For Level I diagnosis, the MDS Task Force proposes impairment on tests of global cognitive ability which are validated for use in PD, such as the Montreal Cognitive Assessment, the Parkinson's Disease-Cognitive Rating scale, Scales of Outcomes of Parkinson's disease-Cognition and the Mattis Dementia Rating Scale [16]. Examples of appropriate neuropsychological testing for each cognitive domain are also now provided within the MDS guidelines [16]. 

## 5. Patterns of Cognitive Deficit in Mild Cognitive Impairment in Parkinson's Disease

Cognitive domains typically affected in PD include executive function, attention, processing speed, visuospatial, learning and memory. A recent review by the MDS Task Force found that PD-MCI may affect a range of cognitive domains, but single domain impairment is more common than multiple domains and within a single domain, nonamnestic impairment is more common than isolated amnestic deficits [15].  Table 1 summarises the prevalence and patterns of PD-MCI in some key cross-sectional studies, for further review see [15]. 

A range of cognitive domains are impaired in PD-MCI. Cognitive deficits in PD have typically been classified as subcortical in nature, though a variety of deficits have been demonstrated including executive, visuospatial, attentional, memory, and language abilities (for review see [15]). Much of the prevalence observed in the nonamnestic single domain may reflect the high proportion of visuospatial and executive function deficits observed in PD, such as problem solving and working memory. Attentional-executive dysfunction is common in most PD patients without dementia and up to half of these may also experience visuospatial and free-recall memory problems [19, 20] whilst memory/learning and language are relatively spared [3, 21]. 

## 6. Predictors of Mild Cognitive Impairment in Parkinson's Disease

PD-MCI is associated with increasing age [19, 20, 22], male gender [20], and lower levels of education [23]. In addition, PD-MCI is generally associated with later onset of disease [20, 22], greater PD severity [19, 20, 22], and longer disease duration [3, 22]. Although not the focus of these studies, increasing dopaminergic medication used by patients with more severe disease may have been a potential confound to the results.

Whilst PD-MCI is associated with more severe motor symptoms in general, it is not entirely clear whether it is associated with a particular motor phenotype. Whilst the majority of studies have reported that nontremor features of bradykinesia, rigidity, postural instability, and gait disorder are particularly associated with cognitive dysfunction [22, 24, 25], some authors have reported that patients with tremor-dominant features are more cognitively impaired [23] and others have failed to identify any difference in motor phenotype [26]. It should be highlighted that these studies did not specifically explore the issue of PD-MCI but focused on the cognitive impairments associated with motor phenotype, although Verbaan et al. (2007) gave specific consideration to the stratification of patients by their age of onset (≤50 and >50) and disease duration (≤10 years and >10 years) [22]. 

The development of PD-MCI has been associated with a number of other nonmotor features, including rapid eye movement sleep behaviour disorder [27], increasing severity of daytime sleepiness [19], and greater autonomic impairment [22]. Furthermore, some authors have found that affective and psychotic symptoms are associated with cognitive impairment [22, 24], though this finding has not been universal with features such as depression [20] and again, such studies were not exploring these features with respect to PD-MCI specifically (i.e., as opposed to cognitive decline in general). 

## 7. Mild Cognitive Impairment and the Transition to Parkinson's Disease Dementia

While current longitudinal data is somewhat limited by the absence of age-matched control groups, PD-MCI patients are at increased risk of dementia compared with cognitively intact patients. This suggests that subtle cognitive decline may represent an early manifestation of dementia. Over short follow-up periods, nondemented patients may display relatively little cognitive decline [28]. However, older age and lower educational attainment amongst PD patients predicts greater decline across global cognitive ability and memory [28]. One four-year follow-up study in prevalent cases found that 62% of PD-MCI patients, compared with only 20% of patients without cognitive deficits, had developed PD-D [29]. Strong predictors of PD-D included single domain nonamnestic MCI, multiple domain MCI, and early impairment on executive function testing [10, 29]. Observing newly diagnosed patients over a period of three and a half years revealed similar proportions who demonstrated cognitive impairment on at least one neuropsychological test (baseline 62%, follow-up 67%) [9]. However, 10% of this study cohort had gone on to develop dementia, an annual incidence of 30 per 1000 person years. Older age, nontremor dominant motor phenotype, higher Unified Parkinson's Disease Rating Scale motor score and poorer performance on semantic fluency, pentagon copying, spatial recognition memory, and the Tower of London planning task predicted a more rapid rate of cognitive decline [9]. In particular, posterior cortical cognitive deficits were suggested as increasing the risk of developing PD-D [9].

The mean time from PD onset to PD-D is approximately 10 years [4, 30], but this varies widely, with some developing dementia much sooner and others remaining free from dementia for 20 years or more [30]. Therefore, a greater understanding about the development of PD-MCI and evolution of PD-D may afford greater prognostic accuracy and more targeted interventions. 

## 8. Biomarkers of Mild Cognitive Impairment in Parkinson's Disease

The ability to correlate cognitive deficits with reliable biomarkers has significant implications in clinical practice, especially in the evaluation of potential neuroprotective therapies. Ideally, biomarkers utilising techniques such as genotyping, neuroimaging, electroencephalography (EEG), and the analysis of cerebrospinal fluid (CSF) should have high sensitivity and specificity, as well as clinical validity when assessing the presence of PD-MCI and progression to PD-D. It is important to note that whilst many studies have not specifically focused on populations of patients with PD-MCI, they are of great utility in improving the understanding of the pathophysiology of PD-MCI. 

### 8.1. Genotyping

There are several genetic influences that are known to impact upon cognitive function in PD, although many have not been evaluated with reference to PD-MCI specifically. The catechol-O-methyltransferase gene contains a functional polymorphism (COMT Val^158^ Met) where the methionine (met) allele has been shown to have a fourfold reduction in enzymatic activity compared with the valine (val) allele [31]. This COMT Val^158^ Met functional polymorphism has been shown to influence executive performance in PD with homozygous met alleles correlating with poorer performance on planning tasks [32]. This poorer executive function has been attributed to a relative hyperdopaminergic state in the dorsolateral prefrontal cortex compared to the striatum [32]. Indeed, recent functional magnetic resonance imaging (MRI) studies have also established that met/met PD patients have a significant reduction in blood oxygen level-dependent signal across the frontoparietal network involved in planning [33] and attentional control [34].

Despite the influence of COMT genotype on executive performance, it does not appear to influence the development of dementia at five-year followup [35]. By contrast, an inversion polymorphism containing microtubule associated protein tau (MAPT, H1 haplotype) has been strongly associated with the development of PD-D [36, 37]. The expression of this gene is believed to be important for maintaining neuronal integrity, but its specific role in the development of PD-D has not been elucidated. 

### 8.2. Neuroimaging

Previous studies have demonstrated that structural MRI may be used to identify and evaluate PD-MCI, although small sample sizes and differences between cognitive assessment methods currently limit the conclusions that may be drawn. Whilst voxel-based morphometry (VBM) seems able to distinguish the pattern of cortical atrophy that exists between patients with PD-D from those with Dementia with Lewy Bodies [38], there appears to be a more continuous spectrum of atrophic change occurring across PD patients with and without cognitive deficits. Comparison between healthy age-matched controls and those with PD-D has generally revealed significant neocortical and hippocampal volume loss, but differentiating distinct changes associated with PD-MCI has been less clear [39, 40]. When compared with PD patients with normal cognition, PD-MCI patients demonstrated hippocampal atrophy and PD-D patients demonstrated hippocampal and additional medial temporal lobe atrophy [41]. PD-MCI patients displayed a different pattern of atrophy to those with normal cognition, and a similar pattern to that of PD-D patients, characterized by atrophy of the hippocampus, prefrontal cortex gray and white matter, occipital lobe gray and white matter, and parietal lobe white matter [41].

Volumetric MRI, even in nonmedicated patients with early PD, has been able to identify some correlations between focal regions of atrophy and specific cognitive impairments [42]. Left hippocampal atrophy was found to be associated with impaired memory, whereas prefrontal cortex atrophy was associated with sustained attention [42]. However, these findings have not been universal and other researchers have failed to find regional grey matter atrophy in newly diagnosed PD patients or any association between grey matter atrophy and cognitive impairment [43]. These conflicting findings assert the pressing need for well-constructed prospective studies to evaluate structural MRI as an early biomarker for dementia. 

In addition to structural MRI, a number of functional neuroimaging approaches have been evaluated in PD patients with cognitive deficits, although again, few have been conducted in those with PD-MCI specifically. Recent work has focused on the use of fluorodeoxyglucose positron emission tomography (FDG-PET) to evaluate cerebral metabolism in patients with PD. This has shown that cognitive deficits are associated with a pattern of decreased prefrontal and parietal metabolism, and increased brainstem/cerebellar metabolism, which increases in severity from those with single to those with multidomain MCI [44]. These altered patterns of metabolism have also been confirmed with magnetic resonance spectroscopy (MRS) in PD. For example, using MRS, Lewis et al. [45] recently reported that a loss of neuronal integrity (measured by N-Acetyl Aspartate [NAA]) within the anterior cingulate in PD was associated with poorer performance on executive functioning measures of set-shifting and response inhibition. Temporoparietal cortical hypometabolism has been found in nondemented PD patients with both P-MRS and FDG-PET [46]. Metabolic changes were detected using MRS measures of NAA/creatine ratio in PD patients, which were correlated with memory [47]. These findings may potentially be used as predictors of cognitive decline; however, work has only been conducted in small cohorts with no longitudinal assessments.

Although, not specifically addressing the evolution of PD-MCI, other studies have been able to correlate executive dysfunction in PD with dopamine levels in striatal and cortical regions utilising fluorodopa PET imaging [48]. Furthermore, reduced levels of Blood Oxygen Level Dependent signal activation have been demonstrated using functional MRI in patients with selective executive dysfunction in both striatal and cortical regions [49], suggesting a possible role for these specific neuroimaging techniques as potential biomarkers in the future studies.

### 8.3. Neurophysiology

In addition to the identification of neuroimaging changes, a number of researchers have reported quantitative EEG characteristics in PD-MCI patients. Increases in the absolute and relative posterior theta amplitude have been noted [50], and in those patients with selective executive dysfunction, an increase in slow wave activity and decreased alpha and fast wave activities at the frontal pole and frontal location have been reported [51]. These observations appear to represent an intermediate electrophysiological state between those patients who are cognitively unimpaired and those with frank dementia, suggesting a possible role for EEG as a physiological tool in the assessment of cognitive decline in PD. 

### 8.4. Cerebrospinal Fluid

Neurochemical biomarkers including CSF and proteomics from blood sampling are also currently being investigated for the assessment of PD-MCI. Much recent work has focused on the analysis of CSF given the possibility that these constituents might indicate components of a degenerative cascade being driven by processes such as oxidative stress or inflammation. One recent prospective study conducted over 12 months demonstrated that a reduced baseline level of CSF amyloid *β* 1–42 (A*β* 1–42) was an independent predictor of cognitive decline in patients with PD [52]. Interestingly, a second study evaluating a cohort of newly diagnosed, untreated patients revealed significant associations between CSF levels of A*β* proteins and memory impairment, but not executive-attentional or visuospatial dysfunction [53]. These findings suggest that alterations in A*β* protein metabolism perhaps acting through the presence of comorbid Alzheimer pathology, may contribute to the heterogeneity in pattern and course of cognitive decline associated with PD. However, decreases in A*β* 1–42 have also been demonstrated in other neurodegenerative disorders lacking distinct plaque pathology [54]. Additionally, in vivo plaque imaging has failed to demonstrate a correlation between plaque load and cognition in PD [55]. Therefore, these findings may suggest a different mechanism of A*β* protein processing, possibly related to synaptic *α*-synuclein pathology [56]. Despite these potential advances, there is some concern that variability between testing laboratories for CSF and more advanced proteomics may limit the utility of such techniques in widespread clinical practice [57]. 

## 9. Treatment of Mild Cognitive Impairment in Parkinson's Disease

### 9.1. Pharmacological Treatment

Considering the significance of PD-MCI, there may be a role for initiating early treatment. Cholinesterase inhibitors have emerged as a potentially useful therapeutic option for cognitive deficits in PD. Cholinesterase inhibitors are widely used in the treatment of Alzheimer's disease, and they have been reported to be beneficial for cognitive impairment in PD and PD-D. In PD patients with dementia or cognitive impairment, Donepezil was associated with improvements in memory, though variable tolerability and side effects warrant careful monitoring [58]. PD-D patients treated with Rivastigmine displayed improved outcomes in the Alzheimer Disease Assessment Scale-Cognitive subscale, though some patients experienced increased frequency of nausea, vomiting, and tremor [59]. A *Cochrane *review based on this trial concluded that Rivastigmine resulted in a clinically meaningful benefit in approximately 15% of cases, with improvements in cognition and activities of daily living [60], though there are significant trade-offs between efficacy and adverse effects [61]. A more recent *Cochrane *review supports the use of cholinesterase inhibitors in patients with PD-D, with a positive impact on global assessment, cognitive function, behavioural disturbance, and activities of daily living; however, the evidence on the use of cholinesterase inhibitors in PD-MCI patients is limited [62]. Additional research is necessary to identify PD patients who may benefit from cholinesterase inhibitors. Furthermore, it is noted that such treatments at best offer symptomatic improvement and do not alter the course or slope of cognitive decline.

Emerging studies have documented benefits from other agents that are purported to have neuroprotective effects. For instance, administering Rasagiline, a selective monoamine oxidase type-B inhibitor, to nondemented PD patients with cognitive impairment may exert beneficial effects on certain aspects of attentional and executive functions [63]. Rasagiline was associated with significant improvements on tests of digit span backwards, verbal fluency, and composite cognitive domain standardised scores in attention, though no benefits were seen in memory, language, and visuospatial functions. 

### 9.2. Nonpharmacological Treatment

Nonpharmacological treatment options may also play a valuable role, and while it has not yet been extensively evaluated in PD, this method of therapy shows promise as a primary and secondary prevention strategy for cognitive decline across a range of neurodegenerative diseases [64]. Cognitive training in nondemented PD patients has been found to improve performance in tests of attention, information processing speed, memory, visuospatial and visuoconstructive abilities, semantic verbal fluency, and executive functions [65]. PD patients who underwent cognitive training have also displayed improvements in performance on the Stroop test and reduced cortical activation patterns visible on fMRI [66]. The authors propose that cognitive training may contribute by promoting brain resources in PD patients, perhaps by readdressing the imbalance caused by alterations to inhibitory circuitry. 

## 10. Conclusion

The widespread acceptance of practice guidelines for the assessment and definition of PD-MCI will hopefully lead to a better understanding of cognitive decline in PD. The accurate characterisation of PD-MCI should identify those “at risk” of developing PD-D and in combination, reliable assessment tools, and biomarkers might allow for the evaluation of future therapeutic interventions. However, further research is required to validate the neurobiology and predictive utility of this diagnostic entity. 

## Figures and Tables

**Table 1:** Prevalence and cognitive profile of PD-MCI.

Authors	Population characteristics	Domains assessed	MCI definition	Prevalence PD-MCI	Cognitive profile found
Aarsland et al., 2009 [67]	Community-based incidental cohort, 196 nondemented, drug-naïve PD patients	Verbal memory Visuospatial ability Attention, executive function	≥1.5 SD below *z* score in at least 1 of 3 domains	18.9%	86.5% SD-MCI 62.2% Nonamnestic 24.3% Amnestic 13.5% MD-MCI 2.7% Nonamnestic 10.8% Amnestic
Foltynie et al., 2004 [32]	Incident cohort of 142 PD patients	Executive function Spatial memory Pattern recognition memory	≥1 SD below normative data in ≥1 test	35.2%	58% SD-MCI 34% Frontostriatal deficits 24% Temporal lobe deficits 42% MD-MCI (frontostriatal and temporal deficits)
Mamikonyan et al., 2009 [19]	106 PD patients, convenience sample with intact global cognition based on age- and education-adjusted MMSE score	Memory Executive function Attention	≥1.5 SD below normative data in ≥1 domain	29.2%	61.3% SD-MCI 29% Attention 19.3% Amnestic 13% Executive 38.7% MD-MCI
Muslimović et al., 2005 [20]	115 newly diagnosed PD patients without “global cognitive deterioration” defined as an MMSE score <24	Executive function Memory Attention Language Visuospatial	≥2 SD below normative data on ≥3 neuropsychological tests	23.5%	MCI subtypes not described

SD-MCI: single-domain mild cognitive impairment. MD-MCI: multiple-domain mild cognitive impairment.
